# The Relationship Between Abnormal Resting-State Functional Connectivity of the Left Superior Frontal Gyrus and Cognitive Impairments in Youth-Onset Drug-Naïve Schizophrenia

**DOI:** 10.3389/fpsyt.2021.679642

**Published:** 2021-10-13

**Authors:** Xiaolei Qiu, Shuiping Lu, Min Zhou, Wei Yan, Jinglun Du, Aoshuang Zhang, Shiping Xie, Rongrong Zhang

**Affiliations:** Department of Psychiatry, The Affiliated Brain Hospital of Nanjing Medical University, Nanjing, China

**Keywords:** youth-onset schizophrenia, ROI, functional connectivity, superior frontal gyrus, cognitive, MCCB

## Abstract

**Objective:** Age of onset is one of the heterogeneous factors in schizophrenia, and an earlier onset of the disease indicated a worse prognosis. The left superior frontal gyrus (SFG) is involved in numerous cognitive and motor control tasks. Hence, we explored the relationship between abnormal changes in SFG resting-state functional connectivity (rsFC) and cognitive function in the peak age of incidence to understand better the pathophysiological mechanism in youth-onset drug-naïve schizophrenia to search for reliable biomarkers.

**Methods:** About 66 youth-onset drug-naïve schizophrenia patients and 59 healthy controls (HCs) were included in this study. Abnormal connectivity changes in the left SFG and whole brain were measured using the region of interest (ROI) rsFC analysis method. The cognitive function was assessed using the MATRICS Consensus Cognitive Battery (MCCB), and the severity of the clinical symptoms was evaluated by positive and negative syndrome scale (PANSS). Furthermore, we analyzed the relationships among abnormal FC values, cognition scores, and clinical symptoms.

**Results:** We found decreased FC between left SFG and bilateral precuneus (PCUN), right hippocampus, right parahippocampal gyrus, left thalamus, left caudate, insula, and right superior parietal lobule (SPL), whereas increased FC was seen between the left SFG and right middle frontal gyrus (MFG) in the youth-onset drug-naïve schizophrenia group, compared with HCs. Meanwhile, the *T*-scores were lower in each cognitive domain than HCs. Moreover, in the youth-onset drug-naive schizophrenia group, the insula was negatively correlated with processing speed. No significant correlations were found between the FC-value and PANSS score.

**Conclusions:** Our findings suggest widespread FC network abnormalities in the left SFG and widespread cognitive impairments in the early stages of schizophrenia. The dysfunctional connectivity of the left SFG may be a potential pathophysiological mechanism in youth-onset drug-naïve schizophrenia.

## Introduction

Schizophrenia is a highly heterogeneous severe mental disorder (1). Age of onset is an important confounding factor, with the peak onset at 16–25 years old. This age group is defined as youth-onset schizophrenia (2–5). A second small peak occurs after the age of 40 years old, referred to as late-onset schizophrenia. Compared with late-onset schizophrenia, youth-onset schizophrenia experiences fewer life changes and stressors essential for the disease's occurrence and development (6). The earlier the age of onset, the more serious the clinical symptoms and cognitive impairments and the heavier the burden to the society and family. Moreover, the neurodevelopment of young schizophrenia patients is incomplete. Hence, the study of schizophrenia at peak ages may provide abundant evidence to understand neurodevelopmental abnormalities in schizophrenia.

Over the past decades, neuroimaging studies have become a hot research area, and the region of interest (ROI) analysis method was widely used in the etiology of schizophrenia (7). Current studies have confirmed that the pathological mechanism of schizophrenia is associated with the abnormality in multiple neural circuits and networks, including default mode network (DMN), salience network (SN), frontoparietal network (FPN), central executive network (CEN), and frontostriatal-thalamic circuit (8–11). However, previous studies mainly focused on chronic schizophrenia patients. Few studies have targeted specific age groups without considering the effects of drugs and disease progression (8–10). Different ages of onset, medication, and disease duration may lead to different brain structures and function damage patterns.

The application of resting-state functional magnetic resonance imaging (rs-fMRI) in elucidating the changes in brain function in specific dorsolateral prefrontal cortex (DLPFC) subareas [left superior frontal gyrus (SFG)] in the high incidence age group has not been fully explored. The SFG is one of the vital brain regions of the DLPFC. The DLPFC is widely interconnected with almost all cortical and subcortical structures, responsible for cognition and mood (12). There is no doubt that the abnormal resting-state functional connectivity (rsFC) of the DLPFC and other brain regions leads to various mental symptoms and cognitive disorders (13–15). Previous studies mainly focused on the global perspective to understand schizophrenia; however, the results were inconsistent or even opposite (8, 11). Zhou et al. (11) reported decreased FC between the bilateral DLPFC and parietal lobes, posterior cingulate cortex (PCC), thalamus, and striatum, while He et al. (8) found increased connectivity between the SFG and thalamus/caudate. These studies' heterogeneity may be due to differences in small sample size, use of drugs or not, course of the disease, age at onset, and selection of seed points.

Numerous studies have confirmed cognitive impairment in schizophrenia patients, involving various cognitive fields (16–18). Cognitive impairments were regarded as core features, existed at each stage of the disease, played a significant role in the prognosis of schizophrenia, and considered a potential treatment target (19–21). Recent studies have shown that the frontal cortex's abnormal functional connectivity was related to cognitive function in schizophrenia, both anatomically and functionally. An anatomical study suggested that PFC–thalamus' decreased connectivity was associated with impaired working memory in schizophrenia but not associated with cognitive flexibility and inhibition (22). Another study found that functional connectivity between the DLPFC and task-related brain regions was significantly impaired. The study also found significant correlations between functional connectivity of the DLPFC and cognitive performance, behavioral disorder, and overall function (23). However, previous studies' sample size was smaller, and the sample homogeneities were lower; hence, the results were unclear. Moreover, there is no study on the relationships between the FC abnormal brain regions of the left SFG and MATRICS Consensus Cognitive Battery (MCCB) in youth-onset drug-naïve schizophrenia.

Hence, based on previous studies' inadequacy, in this study, we used ROI imaging methods and MCCB neurocognitive measurement tools to explore the relationships among abnormal functional connectivity signal value, cognitive impairments, and clinical symptoms. It was hypothesized that (1) compared with a healthy control (HC) group, patients with youth-onset drug-naïve schizophrenia would show abnormal functional connectivity of the left SFG; (2) the cognitive function of the schizophrenia group decreased significantly in various cognitive domains; and (3) we also explored whether the brain areas with abnormal left SFG FC were related to cognitive and clinical symptoms.

## Methods

### Participants

A total of 66 youth-onset schizophrenia subjects and 59 HCs were involved in this study. All the youth-onset schizophrenia patients did not take any antipsychotic drugs, and the course of the disease was ≤24 months. Neuroimaging data were stemmed from the Affiliated Brain Hospital of Nanjing Medical University, Jiangsu, China, from September 2015 to December 2019 (Table 1). Diagnosis of schizophrenia was confirmed by two experienced psychiatrists using the Structured Clinical Interview according to the DSM-5 criteria (24). Healthy controls (59) were recruited through advertising in the local community. The following inclusion criteria apply to both groups: Han people, right-handed, aged 16–25, and able to understand survey instructions and execute cognitive tests. The general exclusion criteria included intellectual disability, head injury, substance abuse or dependence, pregnancy, modified electroconvulsive therapy (MECT) or transcranial magnetic stimulation therapy (rTMS), other neuropsychiatric disorders, or contraindications of MRI. These research procedures were in line with the provisions of the review committee of the Affiliated Brain Hospital of Nanjing Medical University. Written informed consent was obtained from all participants before the study.

**Table 1:** Demographic data, the MCCB scores, IQ, and clinical information in patients and HC.

**Variable**	**SZ (*n* = 66)**	**HC (*n* = 59)**	**Statistics (*t*/*x^**2**^*/*F*)**	***p*-value**
Age (years, mean ± SD)	19.95 ± 2.703^*^	21.59 ± 2.943	*t* = −3.230	*P =* 0.002^a^
Gender (male/female)	45/21	32/27	*x*^2^ *=* 2.561	*P =* 0.110^b^
Education (years, mean ± SD)	12.58 ± 2.198^*^	14.17 ± 2.711	*t* = −3.583	*P =* 0.010^a^
Duration of illness (months)	11.35 ± 7.796	NA	NA	NA
**PANSS**				
Positive symptoms	22.50 ± 3.647	NA	NA	NA
Negative symptoms	20.83 ± 4.037	NA	NA	NA
General	44.59 ± 4.268	NA	NA	NA
All totals	87.85 ± 9.147	NA	NA	NA
**Cognitive domains**				
Speed of processing	35.61 ± 12.381	51.2 ± 8.062	*F* = 59.278	*P* < 0.001
Attention/Vigilance	34.74 ± 10.562	47.29 ± 7.197	*F* = 32.595	*P* < 0.001
Working memory	34.74 ± 8.526	44.32 ± 6.637	*F* = 26.859	*P* < 0.001
Verbal learning	34.98 ± 11.850	47.22 ± 9.512	*F* = 36.613	*P* < 0.001
Visual learning	39.58 ± 11.555	50.02 ± 7.210	*F* = 30.858	*P* < 0.001
Reasoning/problem solving	44.89 ± 12.024	53.93 ± 6.721	*F* = 33.119	*P* < 0.001
Social cognition	34.39 ± 11.290	40.59 ± 10.301	*F* = 10.651	*P* = 0.001
Overall composite	28.91 ± 12.806	46.44 ± 7.548	*F* = 80.651	*P* < 0.001
**IQ**	105.30 ± 11.140	117.64 ± 9.423	*t =* −6.645	*P* < 0.001^a^

*Data are shown as mean ± SD. Compared with the HC group, the patient group showed a significant difference in age and education (*

* *p < 0.05). The scores of seven cognitive domains in the patient groups were significantly lower than in the HC group. SZ, schizophrenia; HC, healthy control; MCCB, MATRICS Consensus Cognitive Battery; IQ, intelligence quotient; PANSS, positive and negative syndrome scale; NA, not applicable*.

a *Two-sample t-test*.

b *Chi-square test*.

### Neuropsychological and Intelligence Quotient

The Chinese version of the MCCB was implemented by two well-experienced psychiatrists to evaluate cognitive function (25, 26). The MCCB is sensitive to the degree of cognitive impairment in seven domains, including speed of processing, attention/vigilance, working memory, verbal learning, visual learning, reasoning, problem solving, and social cognition (25, 26). Each domain's raw scores were corrected by age, gender, and education to get *T*-scores. The Wechsler Adult Scale of Intelligence (WAIS) was used to evaluate the intelligence quotient (IQ). The severity of mental symptoms was assessed by the positive and negative syndrome scale (PANSS) (27), which includes a positive, negative, and general psychopathology scale (27).

### Image Data Acquisition

Neuroimaging was conducted by Germany Siemens 3.0 T signal scanner at the Department of Radiology, Nanjing Brain Hospital, China. All subjects were asked to relax and stay awake and still, and think nothing. Echo planar imaging (EPI) was used to acquire the BOLD-fMRI images. Details of our scanning parameters are provided in Supplementary Table 3.

### Data Preprocessing and Processing

Data Processing Assistant for Resting-State fMRI (DPARSF4.4) advanced edition (http://rfmri.org/DPARSF) was used to preprocess all imaging data (28). The imaging data were calculated in an original space warped by diffeomorphic anatomical registration through exponential Lie algebra (DARTEL). The first four images were discarded for each participant to reduce the influence of noise and magnetic field signal instability, and the remaining imaging was corrected for the acquisition time delay between slices. All subjects with head movement translation >2 mm or rotation angle more than 2° in any direction were excluded (29). Then, functional images were normalized to the Montreal Neurological Institute (MNI) standard space, and the data were resampled in voxels of 3 ×3 × 3 mm size. Low-frequency filtering (0.01 Hz < *f* < 0.08 Hz, TR = 2 s) was employed to remove the high-frequency drift after removing covariate (whole brain signal, cerebrospinal fluid, and movement) interferences. Finally, the resulting imaging underwent spatial smoothing using 4 × 4 × 4 mm as a Gaussian smoothing kernel to improve the signal-to-noise ratio.

### ROI Selections

According to our previous study, meta-analysis results of regional homogeneity (ReHo) abnormal brain changes reported MNI coordinates between schizophrenia and HCs. We chose the left SFG (*x* = 0, *y* = 36, *z* = 48; radius: 6 mm) as the seed point and created FC brain maps with whole brain (see Supplementary Material for details).

### Functional Connectivity and Statistical Analysis

The left SFG was selected as seed point and DPARSF software was used to perform functional connectivity analysis. First, the time series of the left SFG were extracted, and correlation analyses were conducted with the average time series of other voxels in the whole brain to acquire a correlation coefficient (*r*-value). Then, the left SFG functional connection network map of each individual was obtained. Next, Fisher *Z* transformation was used to convert *r*-value into *z*-value to improve normal distribution, thus getting the zFC map of the whole brain for all participants. Finally, zFC-values were used for statistical analysis.

Two-sample *t*-test was performed using DPABI software (http://rfmri.org/dpabi) to find the FC abnormal brain areas between the youth-onset drug-naïve schizophrenia group and HC group. Age, gender, years of education, and frame-wise displacement (FD) were used as covariates, and threshold-free cluster enhancement (TFCE) method was used for multiple comparison correction, and the significance difference level was *p* < 0.05 and the cluster size >14 voxels.

Statistical Package for the Social Sciences version 25.0 (SPSS 25.0) was used for statistical analysis. The two-sample *t*-test was used to compare the difference of demographics and IQ between youth-onset schizophrenia and HCs. Chi-squared test was used for gender. The analysis of covariance was used to analyze the neuropsychological date after taking IQ as a covariate control. Furthermore, Pearson's correlation analysis was performed to examine whether our FC findings were related to cognition and clinical symptoms.

## Results

### Demographic and Clinical Characteristics

**Figure 1:**
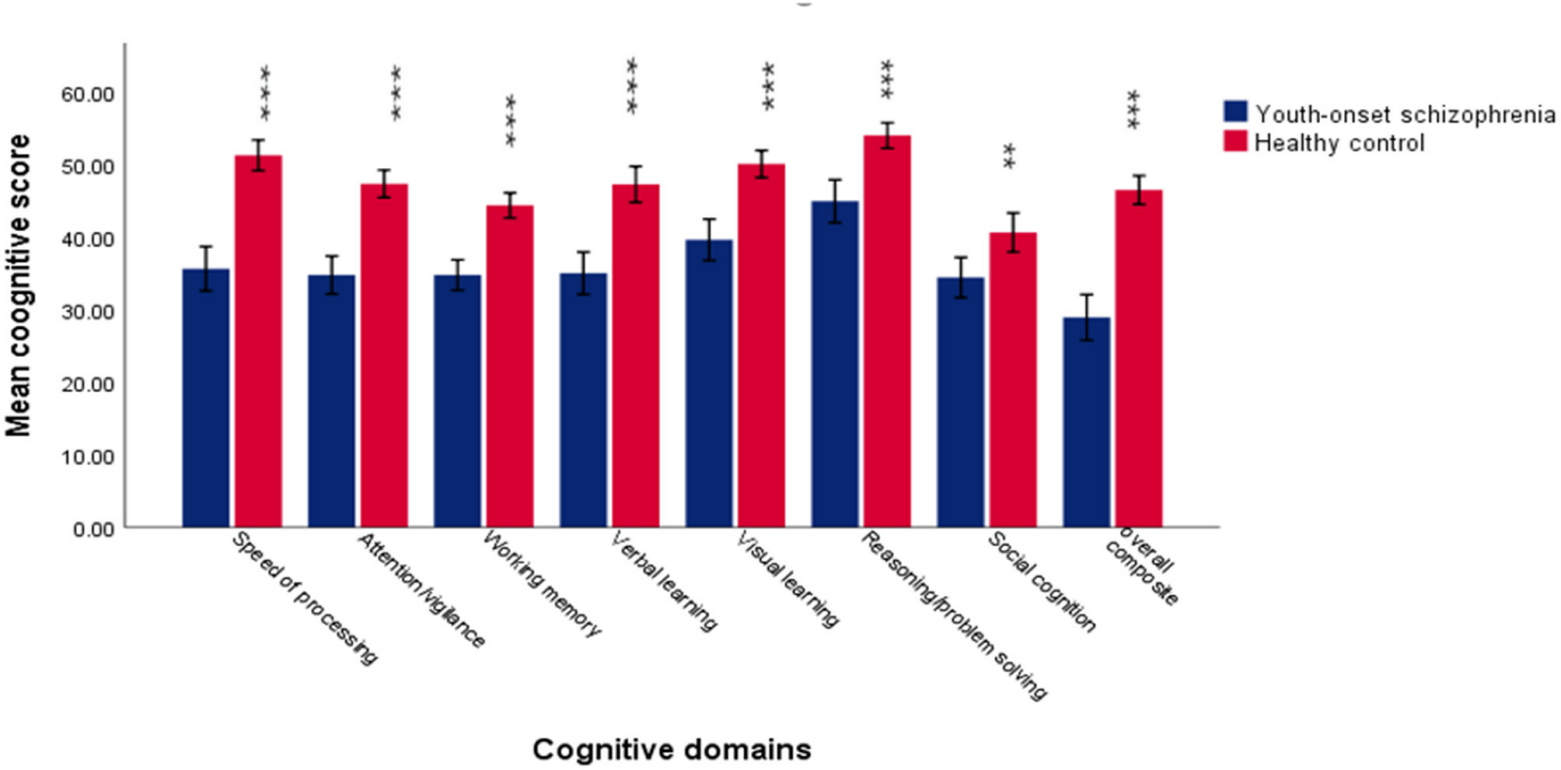
The difference in each cognitive domain between youth-onset schizophrenia and healthy control. The results suggested that patients exhibited significantly lower scores on all the cognitive domains of MCCB. ***Represent *p* < 0.001. **Represent *p* < 0.05. Error bars represent standard deviation.

The demographic and clinical characteristics are displayed in Table 1 and Figure 1. There were significant differences in age (*p* = 0.002), education (*p* = 0.01), and IQ (*p* < 0.001) between the two groups (youth-onset schizophrenia group and HC group) and no significant difference in gender (*p* = 0.11). Considering the cognitive level can be influenced by IQ, the effects of IQ were added to analyze model as a covariate to compare the differences in cognitive domains between the two groups. Compared with HCs, the youth-onset schizophrenia group demonstrated lower scores in each cognitive domain than HCs, especially when processing speed was more obvious.

### Functional Connectivity Network

**Figure 2:**
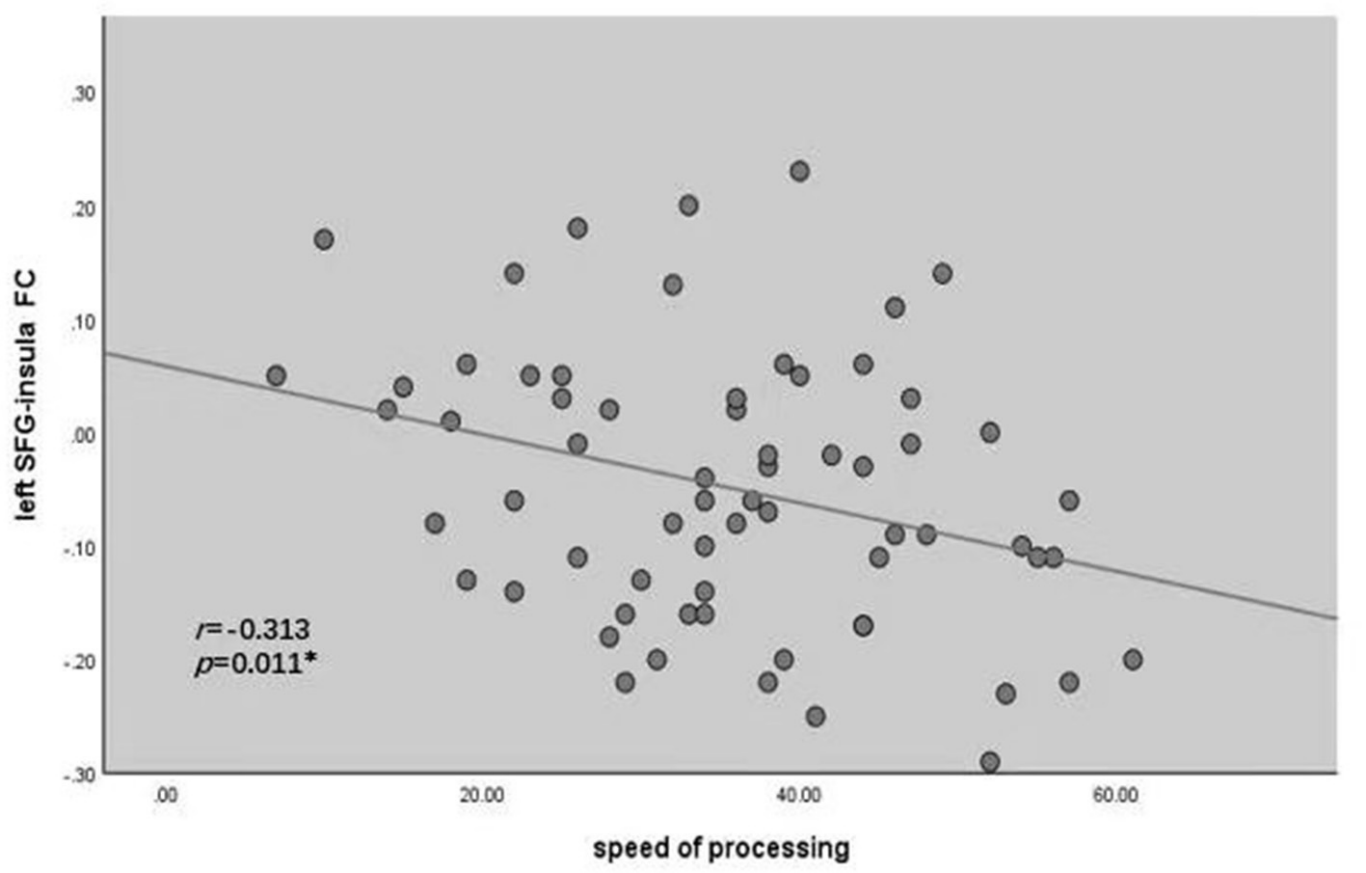
Correlations between the FC of altered regions and cognitive assessments in youth-onset schizophrenia. Pearson's correlation analysis in the patients showed that the decreased FC between left SFG and insula was negatively correlated with processing speed (*r* = −0.313, *P < 0.05). SFG, Superior Frontal Gyrus; FC, functional connectivity.

**Figure 3:**
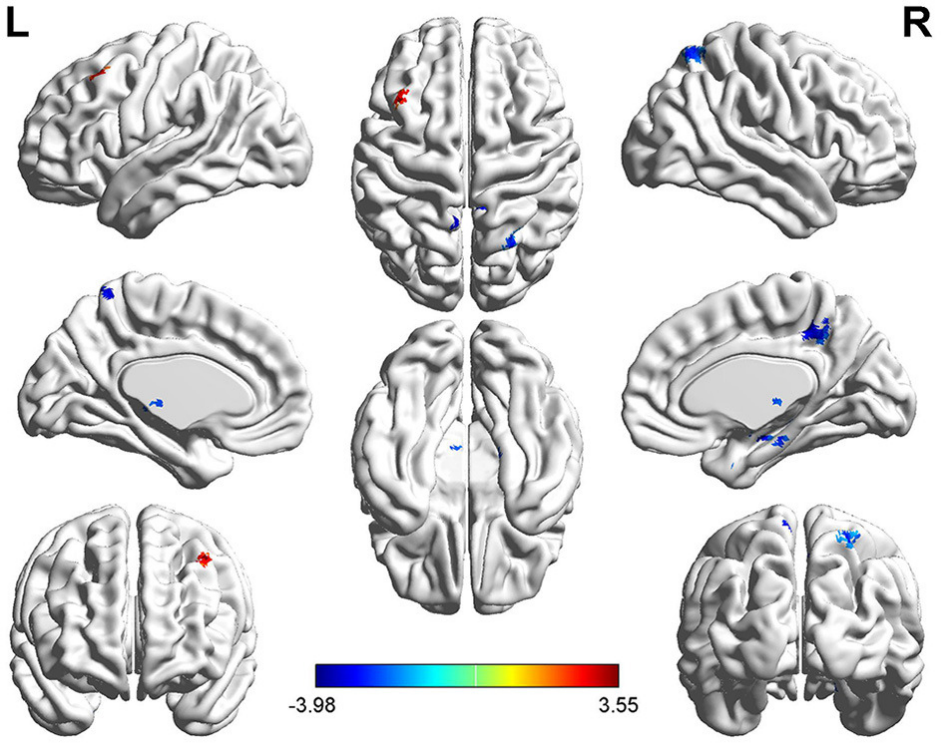
Functional connectivity analysis based on the seed of left SFG between youth-onset schizophrenia and healthy control. The color bar represents T-scores. Warm color represents regions where youth-onset schizophrenia had higher FC, and cold color represents lower FC than HCs. The statistical threshold was set with a combination of voxel-level *p* < 0.05 and cluster size >14 mm^3^ voxels for left SFG FC analysis (youth-onset drug naïve schizophrenia vs. HCs), TFCE corrected after controlling age, gender, education, and frame-wise displacement. L, left; R, Right.

The left SFG functional connectivity outcome is shown in Table 2 and Figure 2. Compared with HCs, results indicated that the youth-onset schizophrenia group showed significantly decreased FC between the left SFG and right hippocampus, right parahippocampal gyrus, left thalamus, insula, left caudate, bilateral precuneus, and right superior parietal lobule (SPL), whereas an increased FC-value in the left MFG was shown (*p* < 0.05, TFCE corrected). Moreover, from Pearson correlation analysis, the insula's FC-value was negatively correlated with processing speed in the youth-onset drug-naïve schizophrenia group (*r* = −0.313, *p* = 0.011, uncorrected, Figure 3). No significant correlations were observed between FC-value and PANSS score.

**Table 2:** Altered regions of left SFG FC based on the ROI analysis between youth-onset schizophrenia and HC.

	**Brain regions**	**Hemisphere**	**Peak MNI**			**Cluster size (voxel)**	***T*-value**
			***x***	***y***	***z***		
SZ < HC							
	Hippocampus	Right	33	−12	−18	88	−3.1232
	Parahippocampal gyrus	Right	21	−24	−18	16	−2.7411
	Thalamus	Left	−3	−21	3	17	−2.5512
	Insula	–	−24	−36	9	26	−3.0971
	Caudate	Left	−15	15	12	46	−3.2242
	Precuneus	Right	9	−42	45	72	−3.9765
	Precuneus	Left	−6	−48	66	35	−3.5159
	Parietal_Sup	Right	30	−66	60	57	−3.4324
SZ > HC							
	Frontal_Mid	Left	−39	21	51	22	3.5538

*SFG, superior frontal gyrus; FC, functional connectivity; ROI, region of interest; MNI, Montreal Neurological Institute; SZ, schizophrenia; HC, healthy control*.

## Discussion

Our study is the first systematic study to explore the relationships between abnormal brain networks of the left SFG and cognitive impairment using the ROI method and MCCB cognitive tool in drug-naïve young schizophrenia. Notably, we used highly homogeneous individuals who were youth-onset, first episode, and without any antipsychotic drugs, which effectively avoid the influence of age at onset and other confounding factors, thus giving us a new perspective to understand schizophrenia. As expected, compared with HCs, the youth-onset drug-naïve schizophrenia group showed significant differences in several neural loops and networks, including the fronto-limbic pathway, fronto-striatal-thalamic-cortical loops, FPN, DMN, CEN, and SN. Each cognitive domain in patients with drug-naïve youth-onset schizophrenia showed poor performance, consistent with a previous study (16). In addition, we identified significant correlations between decreased FC and cognitive domain scores. Our research found that the insula was negatively correlated with the speed of processing. Therefore, the abnormal FC between the left SFG and insula may be the underlying neural basis of cognitive disorder. Regrettably, we found no correlation between abnormal left SFG functional connectivity and clinical symptoms in the patient's group. Future studies with larger sample sizes need to be used for further exploration.

In the present study, we observed decreased connectivity in the bilateral precuneus (PCUN), right hippocampus, and right parahippocampal gyrus associated with left SFG in youth-onset schizophrenia. These regions are the important node of DMN (30), indicating that FC between the SFG and DMN are destroyed in the early stage of schizophrenia. In the resting state, DMN has a strong spontaneous activity closely related to the human brain's monitoring of the external environment, awareness maintenance, emotion processing, self-introspection, and episodic memory extraction (30). Numerous structural and functional imaging studies have confirmed abnormal DMN (31). A disconnectivity functional survey revealed that some brain regions of the DMN in schizophrenia patients, such as the PCUN and part of the frontal lobe, have abnormal functional connectivity (11). The PCUN plays a vital role in regulating the self-consciousness of DMN, a key point of information transmission (30). Evidence from the cognitive model of schizophrenia suggests that the normal interaction disorder between these regions may be the basis of many related symptoms and cognitive deficits (32). Recent studies on the first-degree relatives of patients with schizophrenia found decreased DMN activity in unaffected siblings of schizophrenic patients (33, 34). Moreover, the hippocampus and parahippocampal gyrus also belong to the limbic system. Recent studies have found that the functional connectivity between the medial prefrontal cortex and hippocampus/parahippocampal gyrus was weakened. The parahippocampal gyrus serves as the primary cortical input to the hippocampus, critically involved in cognition and emotion. The hippocampus is considered an emotional and memory hub. Dysfunctional connectivity between the PFC and hippocampus/parahippocampal gyrus in schizophrenia patients leads to dysregulation in working memory (10, 35). Boyer et al. (36) believed that hippocampal dysfunction might cause abnormal long-term memory in patients with schizophrenia, supported by Zhu et al. (37). A meta-analysis of neural correlates of functional outcome in schizophrenia included 37 structural and 16 functional brain imaging studies. A total of 1,631 schizophrenia patients were examined for neural correlation. They found an abnormal alteration in the fronto-limbic system no matter the functional and structural neuroimaging. They suggested that reduced gray matter in the frontal limbic area impairs functional outcomes and leads to extensive cognitive impairment (38). Another study found that compared to HCs, the internal connections between the hippocampus and prefrontal cortex were damaged in first-episode schizophrenia (FES) patients and an at-risk mental state (ARMS) in delayed matching to sample (DMTS) task (39). As described above, this suggested that left SFG integration disorder appeared before the onset of schizophrenia and maybe a potential endophenotype, which provides a basis for early monitoring and early intervention.

Recently, studies about the insula and cognitive impairment are given attention, particularly in the collaborative management of cognitive impairments with the frontal cortex. Salience network anatomy areas include the insula and anterior cingulate cortex (ACC). The insula is an information hub, mainly involved in processing emotion and cognition (40, 41). A *postmortem* study showed a general decrease in myelin and glial density, especially in the white matter region that connects the frontal lobe with the rest of the brain (42, 43). Extensive white matter abnormalities in DTI are associated with the decreased speed of processing (44). Another resting-state FC study found significant correlations between several networks and working memory and processing speed, especially in SN, attention network (AN), and DMN in the schizophrenia group (45). Peter et al. found that the DLPFC-insular connection pathway was related to emotion and cognition; the higher the abnormal degree of DLPFC-insular connection, the more severe the emotional and cognitive impairment (46). In addition, some studies have found that the insula plays a regulatory role in the activation vs. inactivation states of the CEN and the DMN. Therefore, structural and functional changes in the insula may have participated in the onset and progression of schizophrenia (47). In the present study, our main observations were decreased FC between the left SFG and insula. Moreover, a negative correlation between the abnormal FC of the left SFG-insula and processing speed was found. Our findings indicate that lower SFG-insula functional connectivity was related to better cognitive function. These findings suggest that SFG functional connectivity abnormalities may have a negative regulatory mechanism on cognitive function in early schizophrenia. In view of the exploratory nature of the analysis, it should be interpreted with caution.

Compared with HCs, we found decreased connectivity between the left SFG and left caudate/thalamus in youth-onset drug-naive schizophrenia. The caudate nucleus is an important part of the striatum. The lesions of dopaminergic neurons in the striatum may lead to many psychiatric symptoms and cognitive disorders. A multimodal study suggests that dysfunction of the prefrontal cortex in schizophrenia was the cause of dopamine metabolism disorder. Meanwhile, the abnormal relationships between dopamine and the prefrontal cortex may be among the leading causes of schizophrenia (48). The connection between the PFC and thalamus/striatum may control the integration of perception, information representation, and the way people think abstract. Based on an ROI study using DLPFC as seed point to analyze the difference between 17 FES patients and 17 healthy subjects, they found decreased functional connectivity between bilateral DLPFC and the parietal lobe, PCC, thalamus, and striatum in patients with FES, which supported our outcome (11). These results suggest that the intrinsic neuronal activity of the fronto-striatal-thalamic-cortical loops was abnormal in young schizophrenia patients, which may be the pathological mechanism of young schizophrenia. However, some authors failed to replicate this result fully and even found increased FC-value in the thalamus and striatum (8). These reasons may have contributed to the heterogeneity in schizophrenia patients. Age at onset may be an important contributing factor, and another factor may be related to the drug and the disease course. In the future, research on patients of different age groups could help in better understanding schizophrenia and help develop personalized service and management.

Moreover, we observed decreased FC between the SFG and SPL, where increased FC was seen between SFG and MFG. These regions are the essential components of the FPN (49), which is related to memory, language attention, spatial attention, and visual processing (50). A previous study confirmed that schizophrenia patients with abnormal FPN, including functional and structural connectivity, are highly correlated with schizophrenia symptom scores and cognitive function (51). Middle frontal gyrus is a significant part of the PFC involving cognition and emotion management (52). Our previous study confirmed that the MFG was related to attention/vigilance and social cognition (16). The SPL is one of the nodes in FPN, which is related to attention. However, in this study, we did not find any relationship between FPN and cognition, where sample size, sample population, and drug might be the main reasons for the controversy of our findings.

## Limitation

Although our study tries to keep the sample homogeneity and reduce the influence of confounding factors, there are still some limitations. Firstly, this study is cross-sectional, and the causal relationship between abnormal network changes and cognitive function is still unclear. Further longitudinal follow-up studies could answer these questions. Moreover, age and education were not matched between schizophrenia patients and HCs. However, we have corrected age, sex, and education to analyze crude cognitive scores and imaging data analysis. Lastly, the follow-up study will further expand the sample size and apply more rigorous statistical correction methods to explore the relationship between abnormal functional connectivity and cognition in schizophrenia.

## Conclusion

In conclusion, our findings suggest widespread FC network abnormalities in the left SFG and cognitive impairments in the early stages of schizophrenia. The left SFG network contributes significantly to impaired cognition, which may underlie the neuropathological mechanism of schizophrenia, thus further promoting our understanding of frontal cortical dysfunction as a biomarker of cognitive impairments and a neuro-intervention target.

## Data Availability Statement

The original contributions presented in the study are included in the article/supplementary material, further inquiries can be directed to the corresponding author/s.

## Ethics Statement

The studies involving human participants were reviewed and approved by the review committee of the Affiliated Brain Hospital of Nanjing Medical University. Written informed consent to participate in this study was provided by the participants' legal guardian/next of kin.

## Author Contributions

SX and RZ designed the study and revised the manuscript. MZ, WY, JD, and AZ collected the data. XQ and SL analyzed the data and wrote the manuscript. All authors contributed to the article and approved the submitted version.

## Funding

This study was partially supported by the Ministry of Science and Technology of China, National Key R&D Program of China (2016YFC1306805), the Key Project of Nanjing Municipal Bureau of Health Commission (ZKX15033), the General Program of Jiangsu commission of health (H2017051), and the Foundation of Nanjing Medical University (NMUB2020222). These institutions had no role in the design of the study; collection, analysis, and interpretation of data; and writing of the manuscript.

## Conflict of Interest

The authors declare that the research was conducted in the absence of any commercial or financial relationships that could be construed as a potential conflict of interest.

## Publisher's Note

All claims expressed in this article are solely those of the authors and do not necessarily represent those of their affiliated organizations, or those of the publisher, the editors and the reviewers. Any product that may be evaluated in this article, or claim that may be made by its manufacturer, is not guaranteed or endorsed by the publisher.
