# Supplementary material for: The Relationship Between Abnormal Resting-State Functional Connectivity of the Left Superior Frontal Gyrus and Cognitive Impairments in Youth-Onset Drug-Naïve Schizophrenia
Source: Front Psychiatry. 2021 Oct 13;12:679642. doi: 10.3389/fpsyt.2021.679642 (PMC8548582; doi:10.3389/fpsyt.2021.679642)
Supplement: Supplementary file 1 [file Data_Sheet_1.pdf]

1. Supplementary materials

ROI selection: Our previous research on “Regional homogeneity brain alterations in schizophrenia: an activation likelihood estimation (ALE) meta-analysis” has been received but not published. Therefore, we briefly introduce the source of seed points in this paper. We performed a meta-analysis by ALE software (www.brainmap.org) (1) to analyze the difference in Regional homogeneity (Reho) between people with schizophrenia and healthy controls (HCs). First, we searched the literature in four databases (PubMed, Web of Science, Embase, Cochrane) according to the retrieval formula of Supplementary table1. (**Supplementary table1**). A total of 11 studies (2,3,12,4–11) that met the exclusion and inclusion criteria were included (**Supplementary Figure 1**). The coordinates were extracted and synthesized in ALE software. To assure statistical conspicuousness, we used 1000 simulations to test the arrangement of randomly distributed focuses, the cluster level FWE of the ALE map was $p<0.001$ and the cluster-forming threshold was $p<0.001$(13). Finally, compared with the HC group, the schizophrenia group showed decreased ReHo in the bilateral precentral gyrus (PreCG) and left middle occipital gyrus (MOG) and increased ReHo in the left superior frontal gyrus (SFG) (**Supplementary Table 2, Supplementary Figure 2**). We chose the left SFG as the seed point to explore functional connectivity changes in the whole brain.

1. Turkeltaub PE, Eden GF, Jones KM, Zeffiro TA. Meta-analysis of the functional neuroanatomy of single-word reading: Method and validation. *Neuroimage* (2002) 16(3 Pt 1):765-80. doi:10.1006/nimg.2002.1131

2. Wang S, Zhang Y, Lv L, Wu R, Fan X, Zhao J, et all. Abnormal regional homogeneity as a potential imaging biomarker for adolescent-onset schizophrenia: A resting-state fMRI study and support vector machine analysis. *Schizophr Res* (2018) 192:179-84. doi:10.1016/j.schres.2017.05.038

3. Yu R, Hsieh MH, Wang HLS, Liu CM, Liu CC, Hwang TJ, et all. Frequency Dependent Alterations in Regional Homogeneity of Baseline Brain Activity in Schizophrenia. *PLoS One* (2013) 8(3):e57516. doi:10.1371/journal.pone.0057516

4. Shan X, Liao R, Ou Y, Pan P, Ding Y, Liu F, et all. Increased regional homogeneity modulated by metacognitive training predicts therapeutic efficacy in patients with schizophrenia. *Eur Arch Psychiatry Clin Neurosci* (2020). Available at: https://doi.org/10.1007/s00406-020-01119-w (Accessed Mar 25, 2020).

5. Zhao X, Yao J, Lv Y, Zhang X, Han C, Chen L, et all. Abnormalities of regional homogeneity and its correlation with clinical symptoms in Naïve patients with first-episode schizophrenia. *Brain Imaging Behav* (2019) 13(2):503-513. doi:10.1007/s11682-018-9882-4

6. Zhang Y, Guo G, Tian Y. Increased temporal dynamics of intrinsic brain activity in sensory and perceptual network of schizophrenia. *Front Psychiatry* (2019) 10:484. doi:10.3389/fpsyt.2019.00484

7. Gou N, Liu Z, Palaniyappan L, Li M, Pan Y, Chen X, et al. Effects of DISC1 polymorphisms on resting-state spontaneous neuronal activity in the early-stage of schizophrenia. *Front Psychiatry* (2018) 9:137. doi:10.3389/fpsyt.2018.00137

8. Gao B, Wang Y, Liu W, Chen Z, Zhou H, Yang J, et all. Spontaneous activity associated with delusions of schizophrenia in the left medial superior frontal gyrus: A resting-state fMRI study. *PLoS One* (2015) 10(7):e0133766. doi:10.1371/journal.pone.0133766

9. Gao S, Lu S, Shi X, Ming Y, Xiao C, Sun J, et all. Distinguishing between treatment-resistant and non-treatment-resistant schizophrenia using regional homogeneity. *Front Psychiatry* (2018) 9:282. doi:10.3389/fpsyt.2018.00282

10. Liu C, Xue Z, Palaniyappan L, Zhou L, Liu H, Qi C, et al. Abnormally increased and incoherent resting-state activity is shared between patients with schizophrenia and their unaffected siblings. *Schizophr Res* (2016) 171(1-3):158-65. doi:10.1016/j.schres.2016.01.022

11. Cui LB, Liu K, Li C, Wang LX, Guo F, Tian P, et al. Putamen-related regional and network functional deficits in first-episode schizophrenia with auditory verbal hallucinations. *Schizophr Res* (2016) 173(1-2):13-22. doi:10.1016/j.schres.2016.02.039

12. Liu H, Liu Z, Liang M, Hao Y, Tan L, Kuang F, et all. Decreased regional homogeneity in schizophrenia: A resting state functional magnetic resonance imaging study. *Neuroreport* (2006) 17(1):19-22. doi:10.1097/01.wnr.0000195666.22714.35

13. Dehghan M, Schmidt-Wilcke T, Pfleiderer B, Eickhoff SB, Petzke F, Harris RE, et all. Coordinate-based (ALE) meta-analysis of brain activation in patients with fibromyalgia. *Hum Brain Mapp* (2016) 37(5):1749-58. doi:10.1002/hbm.23132

2 Supplementary Figures and Tables

2.1 Supplementary Figures

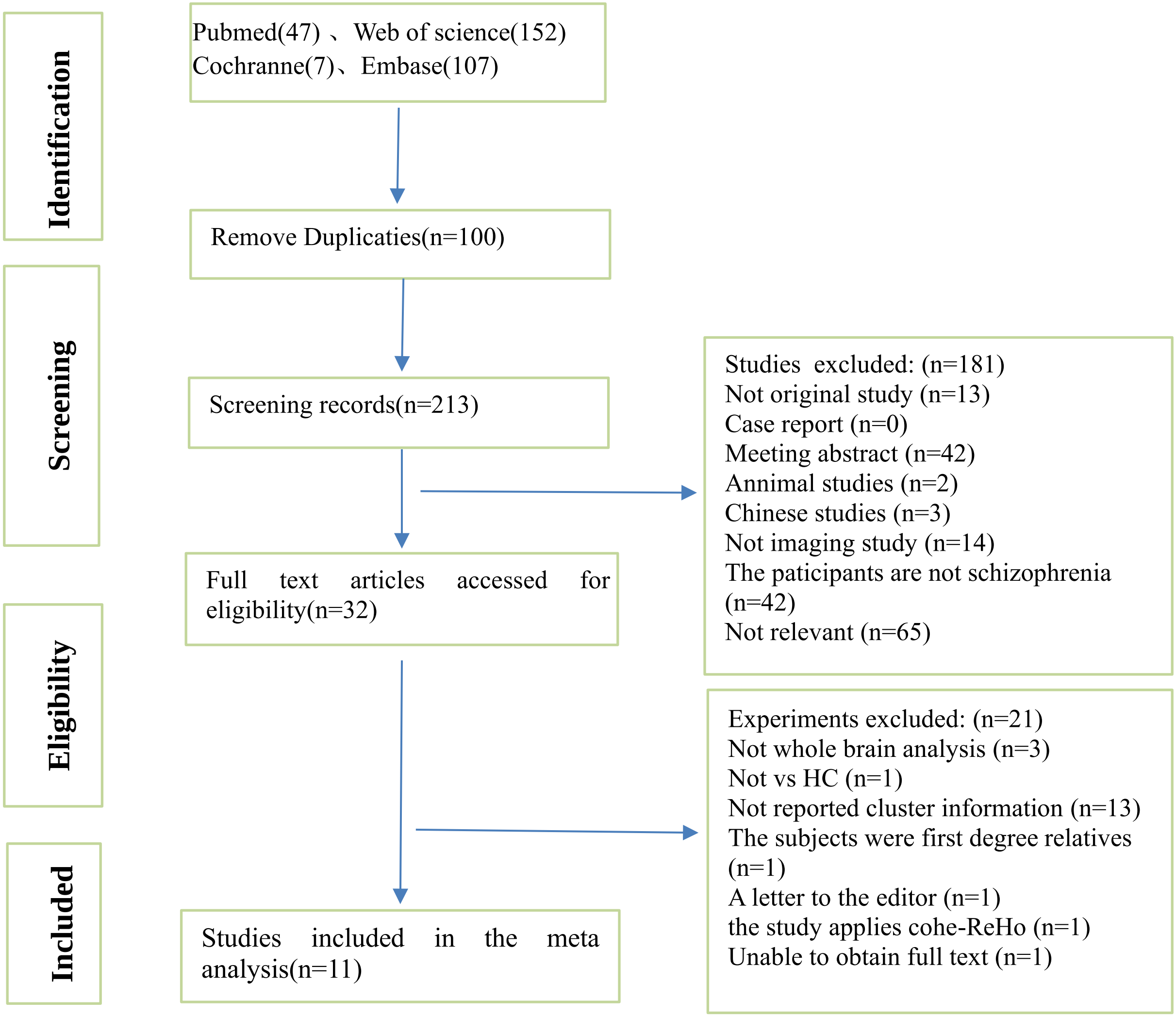

Supplementary Figure 1 Flow-diagram of studies selection

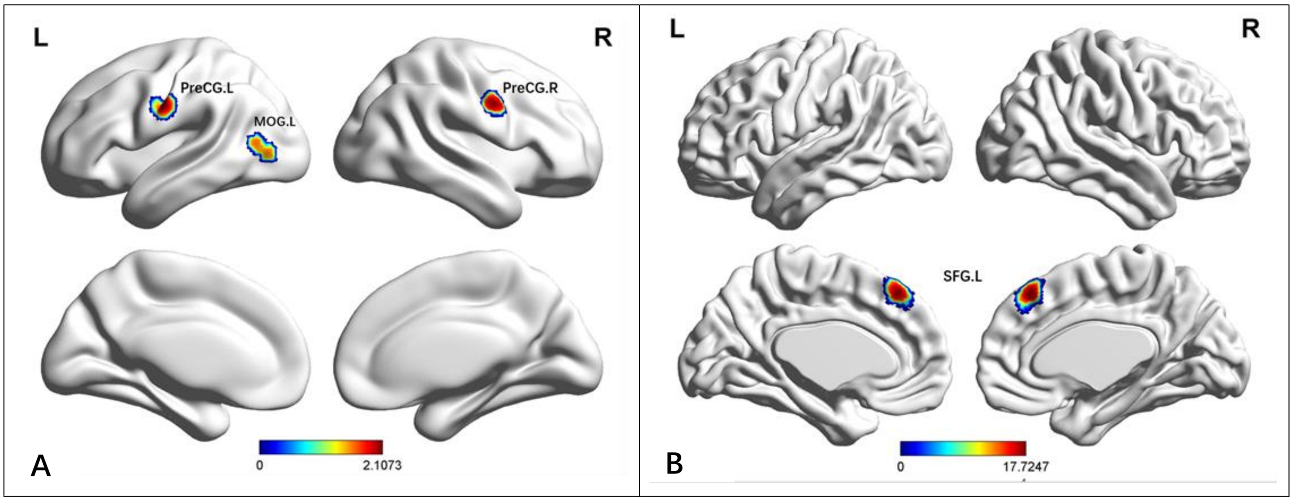

Supplementary Figure 2. (A) decreased ReHo in all schizophrenia patients compared with healthy controls; (B) increased ReHo in all schizophrenia patients compared with healthy controls. Abbreviations: PreCG, precentral gyrus; MOG, middle occipital gyrus; SFG, superior frontal gyrus; R, right; L, left.

2.2 Supplementary Tables

Supplementary Table 1 Search terms for the systematic literature search

**Database**	**Search terms**
PubMed	AllFields:"schizophrenia"[MeSHTerms]OR"Schizophrenias"[Title/Abstract]OR"Schizophrenic disorders"[Title/Abstract]) OR "disorder schizophrenic"[Title/Abstract])OR "disorders schizophrenic"[Title/Abstract])OR "Schizophrenic Disorder" [Title/Abstract])OR "Dementia Praecox"[Title/Abstract]));AND "ReHo"[Title/Abstract]OR "Regional homogeneity" [Title/Abstract]) OR "local consistency"[Title/Abstract])OR"coherence"[Title/Abstract];AND"functional magnetic resonance imaging"[Title/Abstract]OR "fMRI"[Title/Abstract])OR "resting state"[Title/Abstract]
Web of Science	Same as Pubmed
Embase	Same as Pubmed
Cochranne	Same as Pubmed

Supplementary Table 2 Altered ReHo in schizophrenia patients compared with healthy controls

**Cluster**	Cluster size (mm^3)	Local extrema (x, y, z)	Location	Side	ALE value	Brodman
**Decreased ReHo**						
1	2296	–60 –10 34	PreCG	L	0.002360399	4
2	1760	54 –2 34	PreCG	R	0.002120542	6
3	1168	–44 –68 8	MOG	L	0.001864656	37
3	1168	–44 –74 4	MOG	L	0.0017760438	37
**Increased ReHo**						
1		0 36 48	SFG	L	0.0019925283	8

Note: Abbreviations: PreCG, precentral gyrus; MOG, middle occipital gyrus; SFG, superior frontal gyrus; R, right；L, left

Supplementary Table 3 MRI data scanning parameters of all participants

**T1-weighted sagittal images**	**Bold-fMRI images**
repetition time (TR) = 2300 ms	TR/TE = 2500/30 ms
echo time (TE) = 2.96 ms	FOV = 224 mm × 224 mm
inversion time = 900 ms	matrix = 64 × 64
flip angle (FA) = 9°	FA = 90°
field of view (FOV) = 256 mm × 256 mm	slice thickness = 3.5 mm
matrix = 256 × 256	no gap
slice thickness = 1 mm	interleaved transverse slices = 37
sagittal slices = 192	volumes = 149
acquisition time = 554 s	acquisition time = 379 s
